# A Case of Intussusception

**Published:** 1898-08-13

**Authors:** 


### A CASE OF INTUSSUSCEPTION.

### Reduction after Three Weeks' Duration.

So much depends on a due understanding of the length of time during which an intussusception may continue without the formation of such adhesion as to render its reduction impossible, that great interest attaches to a report by Dr. Penrose and Mr. Kellock of a case of intussusception of the cæcum which was reduced with comparative ease after it had existed for three weeks. The case was that of a male child aged sixteen months, who was admitted into the Hospital for Sick Children, Great Ormond Street, on account of pain in the abdomen. Three weeks before admission the mother noticed that the child had pain in the abdomen which came on suddenly, causing him to roll about and double himself up. These attacks had occurred at least once a day, and had been accompanied on three occasions by vomiting. There was no blood in the stools and no straining. The child was strikingly apathetic. In the left iliac fossa a sausage-shaped tumour was felt, lying in the direction of the descending colon and reaching to just below the anterior superior spine of the ilium. It was very hard and somewhat irregular in outline. A less defined tumour could be traced as a continuation of this to the left hypochondrium, across the epigastrium, and down to the middle of the right lumbar region. The right iliac fossa appeared to be more empty than usual. On operation the intussusception was found to be one of the cæcum into itself, the invaginated part reaching the descending colon. Reduction was effected with some difficulty, not, however, from adhesion, but from the thickness and hardness of the gut. No doubt the comparative facility with which reduction was effected was due in large measure to the seat of the mischief, namely, an intussusception of the cæcum into itself. There was thus comparatively little interference with the circulation in the invaginated portions of bowel, as shown by the absence of blood from the stool, and the non-formation of adhesions in the peritoneum. It must be remembered, however, that this is a somewhat rare variety of intussusception, and that the locality of the tumour cannot be always relied on to determine the particular variety of the condition which may be present in a given case.

